# Novel multidrug-resistant sublineages of *Staphylococcus aureus* clonal complex 22 discovered in India

**DOI:** 10.1128/msphere.00185-23

**Published:** 2023-09-12

**Authors:** Monica I. Abrudan, Varun Shamanna, Akshatha Prasanna, Anthony Underwood, Silvia Argimón, Geetha Nagaraj, Sabrina Di Gregorio, Vandana Govindan, Ashwini Vasanth, Sravani Dharmavaram, Mihir Kekre, David M. Aanensen, K. L. Ravikumar

**Affiliations:** 1 Centre for Genomic Pathogen Surveillance, Big Data Institute, University of Oxford, Oxford, United Kingdom; 2 Wellcome Genome Campus, Hinxton, United Kingdom; 3 Central Research Laboratory, Kempegowda Institute of Medical Sciences, Bengaluru, India; 4 Department of Biotechnology, NMAM Institute of Technology, Nitte (Deemed to be University), Mangalore, India; 5 Facultad de Farmacia y Bioquímica, Instituto de Investigaciones en Bacteriología y Virología Molecular (IBaViM), Universidad de Buenos Aires, Buenos Aires, Argentina; Antimicrobial Development Specialists, LLC, Nyack, New York, USA

**Keywords:** *Staphylococcus aureus*, India, WGS, outbreak, ST22, ST239, ST772

## Abstract

*Staphylococcus aureus* is a major pathogen in India causing community and nosocomial infections, but little is known about its molecular epidemiology and mechanisms of resistance in hospital settings. Here, we use whole-genome sequencing (WGS) to characterize 478 *S*. *aureus* clinical isolates (393 methicillin-resistant *Staphylococcus aureus* (MRSA) and 85 methicilin-sensitive *Staphylococcus aureus* (MSSA) collected from 17 sentinel sites across India between 2014 and 2019. Sequencing results confirmed that sequence type 22 (ST22) (142 isolates, 29.7%), ST239 (74 isolates, 15.48%), and ST772 (67 isolates, 14%) were the most common clones. An in-depth analysis of 175 clonal complex (CC) 22 Indian isolates identified two novel ST22 MRSA lineages, both Panton-Valentine leukocidin+, both resistant to fluoroquinolones and aminoglycosides, and one harboring the the gene for toxic shock syndrome toxin 1 *(tst)*. A temporal analysis of 1797 CC22 global isolates from 14 different studies showed that the two Indian ST22 lineages shared a common ancestor in 1984 (95% highest posterior density [HPD]: 1982–1986), as well as evidence of transmission to other parts of the world. Moreover, the study also gives a comprehensive view of ST2371, a sublineage of CC22, as a new emerging lineage in India and describes it in relationship with the other Indian ST22 isolates. In addition, the retrospective identification of a putative outbreak of multidrug-resistant (MDR) ST239 from a single hospital in Bangalore that persisted over a period of 3 years highlights the need for the implementation of routine surveillance and simple infection prevention and control measures to reduce these outbreaks. To our knowledge, this is the first WGS study that characterized CC22 in India and showed that the Indian clones are distinct from the EMRSA-15 clone. Thus, with the improved resolution afforded by WGS, this study substantially contributed to our understanding of the global population of MRSA.

**IMPORTANCE:**

The study conducted in India between 2014 and 2019 presents novel insights into the prevalence of MRSA in the region. Previous studies have characterized two dominant clones of MRSA in India, ST772 and ST239, using whole-genome sequencing. However, this study is the first to describe the third dominant clone, ST22, using the same approach. The ST22 Indian isolates were analyzed in-depth, leading to the discovery of two new sublineages of hospital-acquired *Staphylococcus aureus* in India, both carrying antimicrobial resistance genes and mutations, which limit treatment options for patients. One of the newly characterized sublineages, second Indian cluster, carries the tsst-1 virulence gene, increasing the risk of severe infections. The geographic spread of the two novel lineages, both within India and internationally, could pose a global public health threat. The study also sheds light on ST2371 in India, a single-locus variant of ST22. The identification of a putative outbreak of MDR ST239 in a single hospital in Bangalore emphasizes the need for routine surveillance and simple infection prevention and control measures to reduce these outbreaks. Overall, this study significantly contributes to our understanding of the global population of MRSA, thanks to the improved resolution afforded by WGS.

## INTRODUCTION

Methicillin-resistant *Staphylococcus aureus* (MRSA) is an opportunistic pathogen and a leading cause of community and nosocomial infections, which has prompted the World Health Organization to include it on a list of priority pathogens for R&D of new antibiotics in 2018 (1). Resistance to methicillin is conferred by the *mecA* and *mecC* genes, which are carried on a mobile genetic element, the staphylococcal cassette chromosome mec (SCC*mec*). To date, 15 SCC*mec* cassette types have been defined (2 – 4), but pseudo, composite, and hybrid SSC*mec* cassettes lacking or carrying additional loci are common (5).

MRSA originally appeared in hospitals in the 1960s and then re-emerged in the community and hospitals in the 1980s, spreading worldwide and creating reservoirs in both settings. Traditionally, hospital-acquired MRSA (HA-MRSA) and community-acquired MRSA (CA-MRSA) were considered epidemiologically different clones, as they differed in their antibiotic susceptibility pattern and their mode of acquisition. Moreover, while HA-MRSA mostly affected certain at-risk populations in hospitals, CA-MRSA caused serious illness or even death to otherwise healthy individuals (6). The situation has changed in recent years when CA-MRSA became more invasive and transmissible and increasingly difficult to differentiate from HA-MRSA (7, 8, (9)).

Reports from the second half of the 1990s show that MRSA was endemic in Indian hospitals, and one study which screened 34 healthcare workers reported that more than 50% of these were found to harbor MRSA at any given point (10 , 11). The first genotypic study of *S. aureus* from India, which was conducted between 2003 and 2004 in Bangalore and included 82 isolates (12) and a typing study of random isolates from the Asian Bacterial Bank collected between 1998 and 2003 both identified international clones sequence type (ST) 239 and ST241 as the major circulating STs in hospitals at the time. Subsequent larger studies, conducted after 2004 across multiple sites, found a higher *S. aureus* sequence type diversity, and the major clones were sequence type 22 (ST22), ST772, ST291, and ST239 (68 isolates [13], 292 isolates [14], 412 isolates [15], and 92 isolates [16]). This was confirmed by more recent evidence (study including 300 isolates [17 , 18] and study including 233 isolates [19]), which also highlighted the increasing prevalence of other STs, such as CA-MRSA ST2371 (a single-locus variant (SLV) of ST22), which was first identified in Indian hospitals in 2012 in a tertiary care facility in Mysore, South India (20) (study including 45 isolates). Several of the CA-MRSA clones circulating in India, e.g., ST22-MRSA-IV, ST772-MRSA-V (21), ST672-MRSA-V (17), ST8-MRSA-IV, and ST2371-MRSA-IV (20), carry a smaller SSC*mec* type (IV or V), compared to other, large cassettes (e.g., types I–III) (22) and the Panton-Valentine leukocidin (PVL), a bicomponent toxin encoded by the *lukS-PV* and *lukF-PV* genes residing in a prophage. The PVL toxin causes leukocyte destruction, tissue necrosis, and increased disease severity, and it is thought to contribute to the success of some CA-MRSA lineages (22 , 23).

*S. aureus* ST22-MRSA gained international attention in the late 1990s when the ST22 EMRSA-15 clone began spreading in English hospitals and soon after that became pandemic (24 – 27). Some of the important characteristics of EMRSA-15 were resistance to fluoroquinolones and macrolides and its small SCC*mec* cassette (type IVh) (28). More recently, two other successful ST22-MRSA lineages, genetically distinct from EMRSA-15, were identified in the Gaza Strip (29) and in China (30). The Gaza Strip clone, also found in Russia (31), was sampled from communities of healthy children and adults and was characterized by the *SCCmec* cassette type IVa and an arsenal of toxin genes, including *chp* (CHIPS), *scn* (SCIN), *sak* (staphylokinase), and the toxic shock syndrome toxin-1 (*tsst-1*). The clone found in China was also community acquired, but unlike the Gaza Strip clone, it caused severe bloodstream infections and carried the *SCCmec* cassette type V. Despite the lack of PVL and TSST toxins, its extreme virulence was attributed to elevated expression of the *agr* genes, a quorum sensing system that is a key regulator of *S. aureus* virulence (32).

In the past years, after 2010, another lineage of ST22-MRSA, CA and PVL+, has been reported in countries such as India (33), Kuwait (34), Nepal (35), Japan (36), China (37), and Saudi Arabia (38). Although many studies initially assigned this clone to the European EMRSA-15 ST22 with classic genotyping tools such as multilocus sequence typing (MLST) and pulse-field gel electrophoresis, further analysis of SCC*mec* subtypes and other molecular markers revealed that it was different from the other known ST22 clones and it most likely originated in Asia (38). To our knowledge, the dominant *S. aureus* ST22-MRSA identified in Indian hospitals (by references 33, 15, 13 , 14) has not yet been characterized using whole-genome sequencng (WGS).

In this study, we characterized the Indian *S. aureus* population by WGS of a large retrospective collection of 478 clinical isolates from 17 sentinel sites across 10 states in India collected between 2014 and 2019. Moreover, we harnessed the increased resolution of WGS to contextualize the 175 clonal complex (CC) 22 isolates from India with a global public collection of 1624 CC22 isolates and delineated Indian ST22 lineages distinct from those described previously. Although typing studies are helpful for understanding the current makeup of dominant clones, WGS provides a richer resource for surveillance studies and identification of local outbreaks and facilitates a comprehensive understanding of specific microbes’ dynamics.

## RESULTS AND DISCUSSION

### Population structure of *S. aureus* across 17 sentinel sites in India

To characterize the population structure of *S. aureus* in India, 17 sentinel sites were invited to contribute to MRSA isolates collected from patients between 2014 and 2019 (Table S1). Of the 514 isolates received, 508 were confirmed to be *S. aureus* with Vitek-2 (AST card P628, Biomerieux), and their whole genomes were sequenced. Out of these, 30 genomes did not meet the quality criteria and were further excluded from the study, with the remaining 478 genomes confirmed as *S. aureus in silico*. Out of these, 85 isolates were later characterized as MSSA using phenotypic and genotypic methods (Table S2), and they were also included in the study. The most frequent specimen source was pus (57%, *n* = 274), followed by wound (8.9%, *n* = 43), tracheal (7.7%, *n* = 37), and blood (3.9%, *n* = 19; Table S3). The isolates were collected from patients ranging in age from 4 days to 100 years old (median age 42), and 65.89% (*n* = 315) of the isolates were from males and 34.11% (*n* = 163) were from females (Table S4).

**FIG 1:**
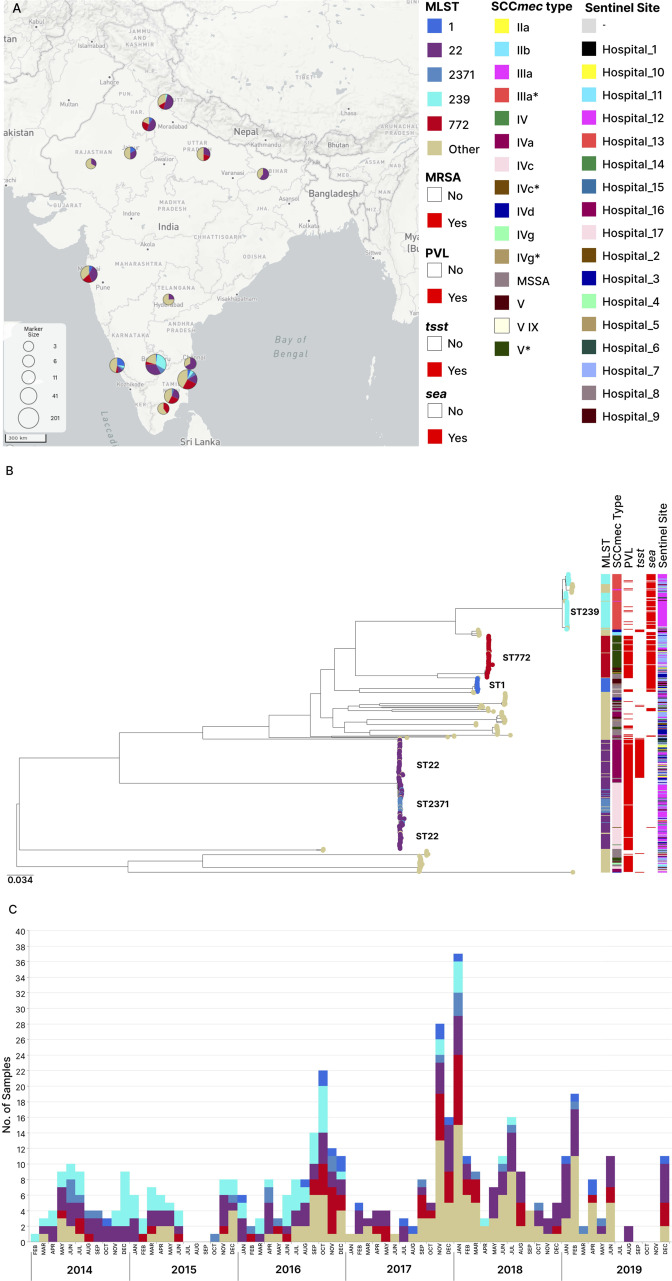
(**A**) Geographic and temporal distributions and main virulence factors of the STs identified in 478 Indian isolates. The map shows the locations of 17 collecting hospitals across 14 different cities. The markers are proportional in size to the number of contributing hospitals in a particular town. For example, Bengaluru, Karnataka, has three contributing hospitals, i.e., hospitals 12, 14, and 6, and hospitals 16 and 4 in Tiruchirappalli, Tamil Nadu. The pie charts show the proportion of the STs identified at each location. (**B**) Phylogeny of the 478 Indian *S. aureus* isolates. The midpoint-rooted phylogenetic tree was inferred from 97,120 informative sites obtained after mapping the genomes to the complete genome of strain *Staphylococcus aureus* MSSA476 (strain GCF_000011525.1 (ST1) and masking regions of recombination and mobile genetic elements. Tree nodes are colored according to their sequence types and are indicated on the map. Scale bars represent the number of single-nucleotide polymorphisms per variable site. Tree nodes are colored according to major STs present in the isolate collection. (**C**) The timeline shows the distribution of samples across the period from February 2014 to December 2019. The bars are colored by ST and are proportional to the number of isolates collected per month. This view is available at https://microreact.org/project/2xDvKQhriNveJ4kiVYsmSQ-s-aureus-wgs-study#i6az-ghru-tree.

The isolates belonged to 35 known STs and 13 novel STs (Tables S5 and S6), and the major clonal complexes were CC22, CC8, and CC1 (Table 1). MRSA isolates mostly belonged to ST22 (*n* = 135), ST239 (*n* = 72), and ST772 (*n* = 59), whereas MSSA isolates belonged to ST1 (*n* = 11), ST672 (*n* = 9), ST2066 (*n* = 9), ST291 (*n* = 7), and ST772 (*n* = 7). The three major STs (ST22, ST239, and ST772) were present across all sampling sites for the entire duration of the study (Fig. 1A through C; Table S5).

**TABLE 1:** A breakdown of the *S. aureus* Indian collection by clonal complexes^*a*^

CC	ST (n)	Count
CC22	**ST22(138**), ST1539 (1), ST2371 (26), ST5092 (1), ST5191 (1), ST6929 (1), ST6930 (1), ST6931 (2), ST6932 (1), ST6933 (1), ST6935 (1), ST6941 (1)	175
CC8	ST8(10), ST72 (3), **ST239**(72), ST368 (14), ST789 (1), ST3324 (1), ST6939 (2)	103
CC1	ST1(21), ST9(3), **ST772 (66),** ST3206 (1), ST6937 (1)	92
Singletons	ST12 (1), ST20 (1), ST88 (4), ST101 (1), ST121 (3), ST291 (7), ST672 (19), ST1156 (1), ST1930 (2), ST2066 (9), ST2233 (3), ST2816 (1), ST2884 (2), ST6934 (1), ST6938 (1)	56
CC30	**ST30 (14),** ST1482 (11), ST6940 (1)	26
CC5	**ST5(10),** ST6(5), ST2689 (8)	23
CC15	ST15 (1), ST6936 (1)	2
CC45	ST508 (1)	1

^*a*^ The most common STs in each clonal complex are shown in bold.

### Antimicrobial resistance (AMR)

*In silico* AMR profiling was performed on 10 antibiotic classes, and the resistance determinants are shown in more detail in Table S7.

One ST239 isolate, sampled from Bangalore in 2015, was found to be resistant to nine classes of antibiotics (G18255029, resistant to all classes tested, except for fusidic acid), while one CC15 isolate, sampled from Pondicherry in 2017, was found to be resistant to only one class of antibiotics (G18255839, resistant to beta-lactams) (Table 2).

**TABLE 2:** Distribution of resistance determinants associated with 10 antibiotic classes across clonal complexes

Antibiotic class	Resistance determinant	Present in CCs (% of samples in that CC which are resistant, n is the number of samples carrying the resistant determinant)	Count (total number of samples carrying the resistance determinants)
Beta-lactams	*blaZ*	CC22 (98%, *n* = 173), CC8 (96%, *n* = 99), CC1 (100%, *n* = 92), singletons (91%, *n* = 51), CC30 (100%, *n* = 26), CC5 (91%, *n* = 21), CC15 (100%, *n* = 2), CC45 (100%, *n* = 1)	465
	*mecA*	CC22 (98%, *n* = 172), CC8 (96%, *n* = 99), CC1 (76%, *n* = 70), CC30 (73%, *n* = 19), singletons (30%, *n* = 17), CC5 (65%, *n* = 15), CC45 (100%, *n* = 1)	393
Macrolides	*ermA*	CC8 (37%, 39), CC30 (4%, *n* = 1)	40
	*ermC*	CC22 (60%, *n* = 105), CC8 (30%, *n* = 31), CC1 (7%, *n* = 6), singletons (11%, *n* = 6), CC5 (22%, *n* = 5), CC30 (12%, *n* = 3), CC45 (100%, *n* = 1)	157
	*msrA*	CC1 (80%, *n* = 74), CC30 (92%, *n* = 24), singletons (18%, *n* = 10), CC5 (35%, *n* = 8), CC8 (4%, *n* = 4), CC22 (0.5%, *n* = 1)	121
Aminoglycosides	*ant(*6*)-Ia*	CC1 (7%, *n* = 6), CC8 (5%, *n* = 5)	11
	*aac(*6*′)-aph(*2″)	CC22 (98%, *n* = 172), CC8 (96%, *n* = 99), CC1 (84%, *n* = 77), singletons (48%, *n* = 27), CC30 (92%, *n* = 24), CC5 (39%, *n* = 9)	408
	*aph(*3′*)-III*	CC1 (89%, *n* = 82), CC8 (50%, *n* = 52), CC30 (92%, *n* = 24), singletons (39%, *n* = 22), CC5 (35%, *n* = 8), CC22 (1%, *n* = 2)	190
	*aadD*	CC1 (*n* = 1), CC5 (1%, *n* = 8), CC8 (0.8%, *n* = 4), CC22 (20.9%, *n* = 100), singletons (*n* = 3)	116
Fluoroquinolones	*grlA_E84K*	CC8 (4%, *n* = 4), CC1 (1%, *n* = 1)	5
	*grlA_S80Y*	CC1(60%, *n* = 55), CC22(1%, *n* = 2), CC30(8%, *n* = 2), singletons (5%, *n* = 3)	62
	*grlA_S80F*	CC1 (38%, *n* = 35), CC15 (0%, *n*=), CC22 (99%, *n* = 173), CC30 (92%, *n* = 24), CC5 (65%, *n* = 15), CC8 (100%, *n* = 103), singletons (89%, *n* = 50)	400
	*grlB_D432N*	CC8 (48%, *n* = 49)	49
	*grlB_P585S*	CC8 (7%, *n* = 7)	7
	*gyrA_G106D*	CC8 (44%, *n* = 45)	45
	*gyrA_S85P*	CC8 (7%, *n* = 7)	7
	*gyrA_S84A*	Singletons (2%, *n* = 1)	1
	*gyrA_S84L*	CC1 (97%, *n* = 89), CC22 (99%, *n* = 174), CC30 (88%, *n* = 23), CC5 (48%, *n* = 11), CC8 (91%, *n* = 94), singletons (89%, *n* = 50)	441
	*gyrA_E88K*	CC8 (5%, *n* = 5)	5
Mupirocin	*ileS_V588F*	CC30 (4%, *n* = 1), CC8 (1%, *n* = 1)	2
	*mupA*	CC8 (4%, *n* = 4), singletons (4%, *n* = 2), CC1 (1%, *n* = 1), CC22 (0.5%, *n* = 1), CC30 (4%, *n* = 1)	9
Tetracycline	*tet(K*)	CC8 (26%, *n* = 27), CC1 (12%, *n* = 11), CC5 (35%, *n* = 8), CC22 (3%, *n* = 5), singletons (9%, *n* = 5), CC30 (8%, *n* = 2)	58
	*tet(M*)	CC8 (86%, *n* = 89)	89
Trimethoprim	*dfrC*	CC22 (88%, *n* = 154), CC8 (27%, *n* = 28), CC5 (35%, *n* = 8)	190
	*dfrG*	CC1 (80%, *n* = 74), CC30 (100%, *n* = 26), CC5 (74%, *n* = 17), CC8 (15%, *n* = 15), singletons (13%, *n* = 7)	139
Fusidic acid	*fusC*	CC1 (18%, *n* = 17), CC5 (17%, *n* = 4), singletons (2%, *n* = 1)	22
	*fusA_L461K*	CC8 (6%, *n* = 6)	6
	*fusA_E444K*	CC1 (1%, *n* = 1)	1
Fosfomycin	*fosB*	CC8 (99%, *n* = 102), CC1 (77%, *n* = 71), singletons (54%, *n* = 30), CC30 (100%, *n* = 26), CC5 (78%, *n* = 18), CC15 (50%, *n* = 1)	248
Rifampicin	*rpoB_H434N*	CC8 (57%, *n* = 59)	59
	*rpoB_L419S*	CC8 (44%, *n* = 45)	45

The most common resistance gene found in the collection was *blaZ*, conferring resistance to beta-lactams (present in 465 of the 478 tested isolates), while the least common resistance gene found was *mupA*, conferring resistance to mupirocin (present in 9 of the 478 tested isolates) (Table 2).

### Identification of a potential persistent ST239 outbreak in one Indian sentinel site using WGS

ST239-MRSA is an invasive, highly recombinant, and virulent clone that causes a wide range of life-threatening infections. It has caused hospital epidemics starting from the 1970s throughout the world (39). It is still one of the dominant clones in Asia (40 , 41) and in Southern India (42 , 43), although it is slowly being replaced by ST22 and ST772 (15). ST239-MRSA clones were found to carry several types of the SCC*mec* cassette, including SCC*mec* types V, III, IV, and I (44). A multidrug-resistant (MDR) ST239-MRSA-III clone exhibiting high resistance to mupirocin and inducible clindamycin resistance has been reported in India since 2010, also carrying the PVL genes (44).

The ST239 isolates were collected from five sentinel sites across the country, and 100%of these were MRSA. All of the ST239 isolates carry a composite version of the SCCmec cassette type III (type III*). We further investigated the structure of the SCC*mec* type III* cassette in a representative ST239 isolate (G18252308, Fig. S1) with nanopore sequencing. SCCmecFinder v.1.2 showed an 83% similarity between the SCC*mec* type III* cassette and the template sequence AB037671.1, found in strain 85/2082 (https://www.ncbi.nlm.nih.gov/nuccore/AB037671.1/). Unlike the template sequence, the full-length SCCmec III* sequence lacks the *mer* operon, responsible for resistance to mercury and the pT181 region, but contains extra genes previously found in SCC*mec* type I, such as the *pls* gene, which encodes the plasmin-sensitive surface protein, a virulence factor involved in septic arthritis and sepsis.

**FIG 2:**
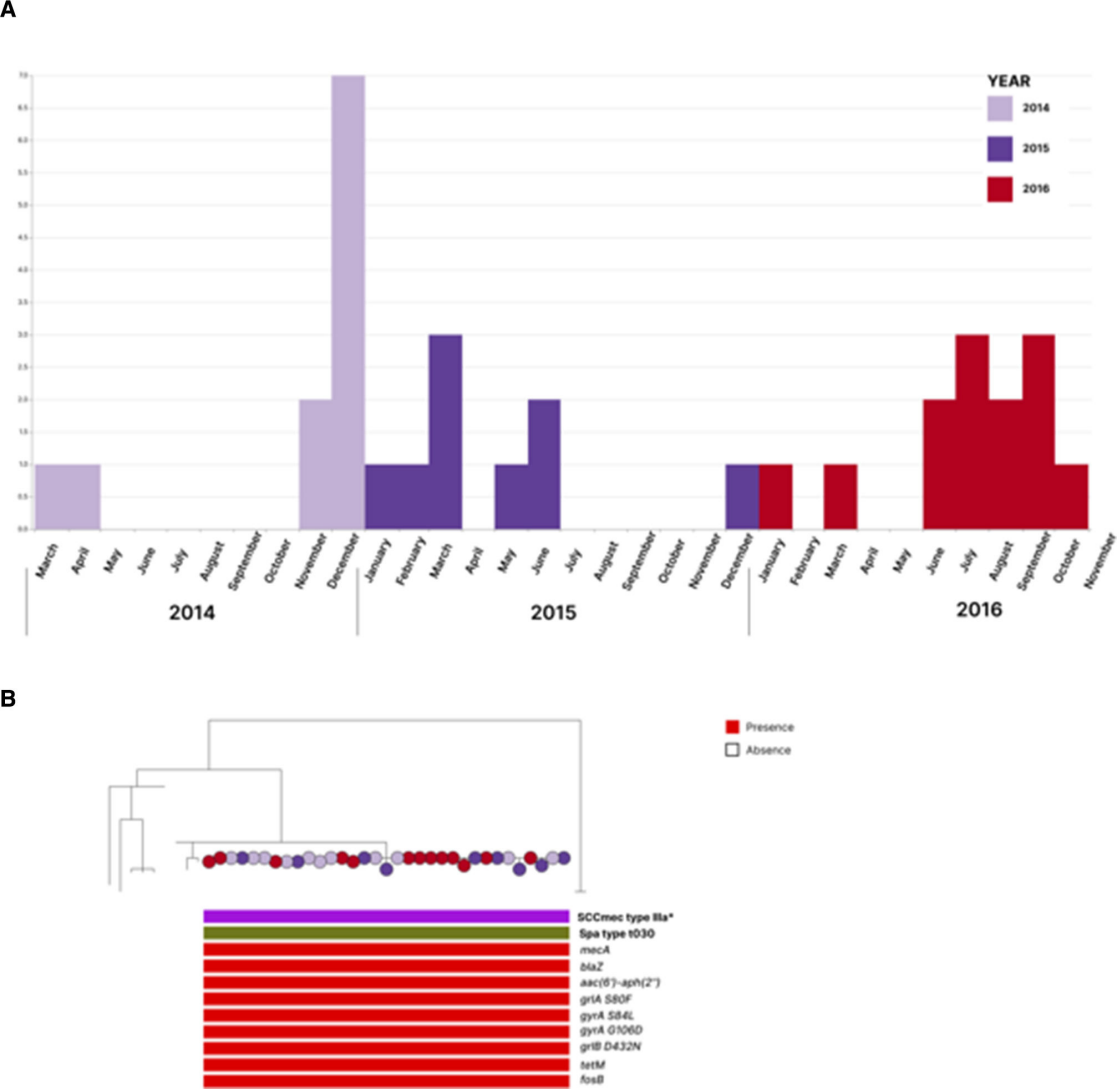
(**A**) The timeline shows the distribution of ST239 samples across the outbreak period from March 2014 to October 2016. The bars are colored year-wise. This view is available at https://microreact.org/project/2xDvKQhriNveJ4kiVYsmSQ-s-aureus-wgs-study#ffxiv-st239-outbreak. (B) Phylogenetic tree, with linked genotypic data of outbreak isolates at one sentinel site in Bangalore. The maximum-likelihood phylogenetic tree of 43 closely related ST239 isolates was generated using *S. aureus* ST239 T0131 GCF_000204665.1_ASM20466v1 as a reference genome, identified based on similarity using BactInspector (46). The central cluster of 33 isolates collected from hospital 12 in Bangalore, between 2014 and 2016, is highlighted, together with information regarding the isolates’ *SCCmec* type, spa type, and AMR genetic determinants. The nodes are colored as per the year of collection. The interactive view is available at https://microreact.org/project/2xDvKQhriNveJ4kiVYsmSQ-s-aureus-wgs-study#yxiv-st239-outbreak-with-genome-t0131-as-a-reference.

Forty-four genomes belonging to ST239 and with identical spa types (*t030*) were aligned to the *S. aureus* ST239 T0131 reference genome (accession GCF_000204665.1_ASM20466v1), and mean pairwise single-nucleotide polymorphism (SNP) differences were computed between isolates. We identified a cluster of 33 isolates collected from the same sentinel site in Bangalore between 2014 and 2016 (Fig. 2A; https://microreact.org/project/2xDvKQhriNveJ4kiVYsmSQ-s-aureus-wgs-study#yxiv-st239-outbreak-with-genome-t0131-as-a-reference), where the mean pairwise SNP differences between isolates were seven SNPs. In contrast, the mean pairwise SNP differences between isolates outside the cluster of 33 isolates were 82 SNPs. Our results suggest a nosocomial outbreak that persisted over a period of 3 years. The phylogeny of the 33 isolates is shown in Fig. 2B. One plausible hypothesis is that this outbreak could have a single contamination source, such as a healthcare professional as in the case of the MRSA outbreak in Cambridge (45). However, in the absence of epidemiological information, we cannot state this for sure.

### Two novel ST22 MRSA clones, both PVL+ and one harboring the *tsst-1* gene

**FIG 3:**
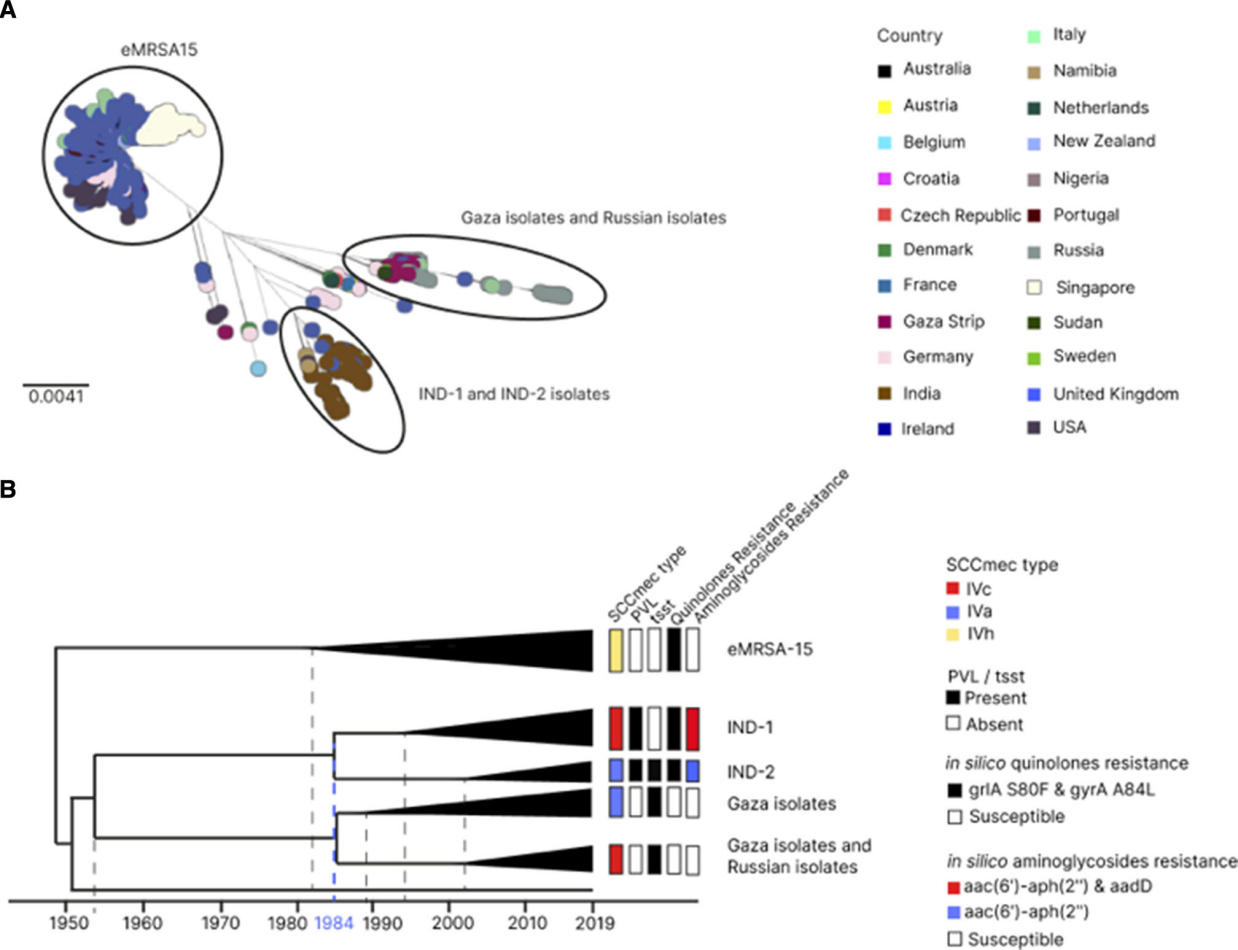
(**A**) Indian CC22 strains in a global context. Phylogenetic tree of the ST22 global collection. The midpoint-rooted phylogenetic tree was obtained from 44,703 SNPs after mapping the genomes to the complete genome of strain EMRSA_15_GCF_000695215 and masking regions of recombination and mobile genetic elements. Tree tips are colored by country of origin as described in the legend. Scale bars represent the number of single-nucleotide polymorphisms per variable site. The interactive views are available at https://microreact.org/project/2xDvKQhriNveJ4kiVYsmSQ-s-aureus-wgs-study#olhe-cc22-global-view-rectangular-tree https://microreact.org/project/2xDvKQhriNveJ4kiVYsmSQ-s-aureus-wgs-study#6w7r-cc22-global-view-radial-tree. (**B**) Bayesian temporal tree of 1621 ST22 Indian and global isolates with estimated ancestral dates. Tree nodes from major ST22 lineages have been collapsed for clarity. The tree is annotated with the *SCCmec* types, the presence/absence of the PVL and *tsst-1* virulence genes, and the predicted resistance to quinolones and aminoglycosides. The MRCA of IND-1 and IND-2 is marked with blue on the timeline. The data used to generate this tree are available at https://microreact.org/project/2xDvKQhriNveJ4kiVYsmSQ-s-aureus-wgs-study#6w7r-cc22-global-view-radial-tree.

Out of the 175 CC22 isolates from this collection, 138 belong to sequence type 22; 26 belong to ST2371; and 11 belong to other STs. In order to better understand the relationships between the Indian isolates, we contextualized them with a large collection of 1623 CC22 isolates from previously published studies (Fig. 3A). Altogether, 1408 genomes belonged to the EMRSA-15 clone and 105 belonged to the “Gaza clone” (Table 3; Table S10). A temporal analysis of the CC22 genomes with BactDating (47) showed that the Indian isolates form one monophyletic group consisting of two distinct subclusters (first Indian cluster [IND-1] and second Indian cluster [IND-2]) (Fig. 3B), with overlapping geographic distributions and with an MRCA in 1984 (95% HPD: 1982–1986). The analysis of the global collection also revealed an isolate from St. Petersburg, Russia, collected in 2019 (RU 1246, accession SRR12560298 [48]) nested within the IND-2 clade (see Microreact view; https://microreact.org/project/2xDvKQhriNveJ4kiVYsmSQ-s-aureus-wgs-study#me32-cc22-ind-2-isolates-with-one-russian-isolate). The isolate from St. Petersburg was reported to be the most divergent in the Russian collection and carried *tsst-1* and the SCC*mec* IVa cassette, which were also observed in the Indian isolates of this study. Moreover, this isolate had the same spa type, t005, as 34 isolates belonging to the IND-2 cluster. Due to our limited metadata on the Russian SRR12560298 isolate, we are unable to definitively determine whether this is a case of international transmission or if the isolate originated from an individual from India travelling to Russia.

**TABLE 3:** Global studies including CC22 genomes used for contextualization of the 175 CC22 genomes in this study

Study name	Bioproject number	Country	Study period	Citation	Publication link	No. of isolates	Invasive/carriage	Description
Aanensen (2016)	PRJEB2478	Nine countries in Europe	2006–2007	(49)	https://mbio.asm.org/content/7/3/e00444-16.short	40	Invasive	*S. aureus* European structured survey
Chow (2017)	PRJEB9390	Singapore	2014–2016	(50)	https://academic.oup.com/cid/article/64/suppl_2/S76/3782668?login=true https://www.sciencedirect.com/science/article/pii/S1198743X20304420	183	Carriage	Nasal, axillary, and groin swabs from patients in healthcare facilities
Reuter (2015)	PRJEB2756	UK	2001–2010	(51)	https://genome.cshlp.org/content/26/2/263	783	Invasive and carriage	MRSA submitted to the British Society for Antimicrobial Chemotherapy by UK laboratories between 2001 and 2010
Chang (2018)	PRJNA484934	Gaza (Palestine)	2009	(52)	https://www.ncbi.nlm.nih.gov/pmc/articles/PMC6113745/	59	Carriage	Nasal swabs from healthy children under 5.5 yr
Manara (2018)	PRJNA400143	Italy	2013	(53)	https://www.ncbi.nlm.nih.gov/pmc/articles/PMC6234625/	14	Invasive	Pediatric hospital
Gostev (2021)	PRJNA609231	Russia (Moscow, St. Petersburg)	2015–2019	(48)	https://www.sciencedirect.com/science/article/pii/S0924857920304842	54	Carriage	Routine screening
Nubel (2013)	PRJNA192952	Germany	2010	(54)	https://journals.plos.org/plosone/article?id = 10.1371/journal.pone.0054898	44	Carriage	Neonatal intensive care unit
Bletz (2015)	PRJEB7089	Germany	2013–2014	(55)	https://www.sciencedirect.com/science/article/pii/S1198743X14000913?via%3Dihub	117	Invasive and carriage	Clinical samples or screening swabs
Hsu (2015)	PRJEB2295	Singapore	2000–2010	(56)	https://genomebiology.biomedcentral.com/articles/10.1186/s13059-015-0643-z	87	Invasive and carriage	Clinical samples
Holden (2013)	PRJEB2096, PRJEB2510	Global	1990–2009	(28)	https://genome.cshlp.org/content/23/4/653.short	150	Invasive and carriage	Global collection
Warne (2016)	PRJEB2755	UK	1998–2012	(57)	https://bmcgenomics.biomedcentral.com/articles/10.1186/s12864-016-2426-7	11	Invasive	Human bacteremia
Lowder (2009)	PRJNA312437	Global	1954–2007	(58)	https://www.pnas.org/content/106/46/19545.full	1	Invasive	Human to poultry host jump
Holmes (2016)	PRJEB2655	Global	1998–2012	(59)	https://pubmed.ncbi.nlm.nih.gov/24825010/	79	Carriage	Human and companion animals
Harris (2013)	PRJEB2737	UK	2011	(45)	https://www.sciencedirect.com/science/article/pii/S1473309912702682	75	Carriage	Hospital outbreak
GHRU-India This Study	PRJEB29740	India	2014–2019	NA	NA	175	Invasive	Clinical samples

### The CC22 IND-1 cluster

The IND-1 cluster is formed of 107 PVL+ isolates, identified in 9 of the 17 contributing sentinel sites. The average pairwise SNP difference between IND-1 isolates is 86 (0–195), and based on *in silico* methods, these isolates were predicted to be resistant to beta-lactams (107), macrolides (50), aminoglycosides (106), quinolones (106), tetracycline (2), and trimethoprim (60). The dominant spa type in this cluster is t852 (82 of 107 isolates).

An analysis of the accessory genomes of Indian ST22 isolates using Panaroo (61) revealed that all genomes in the IND-1 cluster carry a version of the SraP protein, which is 111 amino acids shorter than that found in the genomes from the IND-2 cluster, caused by a deletion in an area of the IND-2 SraP with repeated sequences. SraP is a serine-rich adhesin that mediates binding to human platelets, possibly through a receptor-ligand interaction, and is a potential virulence determinant in endovascular infection (62). Clade-specific versions of the SraP proteins were also identified in a study of the CC30-MRSA from Argentina (63).

The SCC*mec* cassette type IVc found in the IND-1 isolates was 80% similar to the SCC*mec* cassette from the reference genome of strain 81 of 108 (MR108) (Genbank accession AB096217). Using nanopore long-read sequencing, we obtained the complete sequence of the SCCmec cassette IVc from a representative genome (G18255819), which showed the integration of puB110 plasmid carrying the *aadD1* and *bleo* aminoglycoside resistance genes (see Fig. S2) and the different location of the *aacA-aph* gene compared to the reference sequence.

A detailed view of the CC22 IND-1 cluster in a global context (see the Microreact view; https://microreact.org/project/2xDvKQhriNveJ4kiVYsmSQ-s-aureus-wgs-study#sgfb-cc22-ind-1-isolates-in-global-context) shows that this clone had spread to the UK and Italy.

### ST2371, a subclone of the CC22 IND-1 cluster

ST2371 is a single-locus variant of ST22 and a prevalent community-associated MRSA MDR clone. ST2371-MRSA was previously reported in Southern India (16, 20) but also sporadically in other parts of the world (64). The 27 ST2371 Indian isolates from this study were collected from four sentinel sites and are closely related to the other ST22 isolates from the IND-1 clade (the mean pairwise SNP difference between the ST22 and the ST2371 genomes is 85 (minimum [min] 69, maximum [max] 173) (see the Microreact view; https://microreact.org/project/2xDvKQhriNveJ4kiVYsmSQ-s-aureus-wgs-study#v5eu-ghru-st22-isolates-in-global-context). Similar to the 58 ST2371 isolates from a 2011 outbreak in a neonatal unit in Cambridge, UK (45), the ST2371 Indian isolates identified in this study carry the SCC*mec* cassette type IVc and are PVL+. The mean SNP difference between the Cambridge ST2371 isolates and the Indian ST2371 isolates is 54 (min 36, max 76) (see the Microreact view; https://microreact.org/project/2xDvKQhriNveJ4kiVYsmSQ-s-aureus-wgs-study#1osl-st2371-in-global-context).

### The CC22 IND-2 cluster

The second Indian cluster comprised 60 isolates, collected from 10 sentinel sites (Microreact view; https://microreact.org/project/2xDvKQhriNveJ4kiVYsmSQ-s-aureus-wgs-study#me32-cc22-ind-2-isolates-with-one-russian-isolate), with an average pairwise SNP difference between isolates of 96 (0–359).

The dominant spa type in the IND-2 cluster is spa t005 (34 of 60 isolates), and the second most dominant one is spa t309 (12 of 60 isolates).

Unlike isolates from clade IND-1, isolates from clade IND-2 carried the *SCCmec* cassette type IVa; they lack the *aadD* gene conferring resistance to aminoglycosides and harbor the *tsst-1* gene. An analysis of the ST22 pangenome identified the genes *entC1*, *gloB* (uncharacterized metallo-beta-lactamase), and *tsst* as being specific to the genomes in the IND-2 cluster. Enterotoxin type C-1, a product of *entC1*, is a heat-stable enterotoxin produced by *S. aureus* that activates the host immune system by binding as unprocessed molecules to major histocompatibility complex class II and T-cell receptor molecules. Enterotoxin type C-1 is often found in processed food from raw milk and can cause food poisoning, and its presence in dairy products has been used as an indicator of food contamination (65).

Although absent from the EMRSA-15 reference genome, enterotoxin C (66) was also present in the majority of the ST22 EMRSA-15 genomes (the clade originally called ST22-A [28]). When compared with the reference genome EMRSA-15, the ST22 isolates from the IND-2 cluster have a shorter ComF operon protein one than the reference at position 789971, by 273 nucleotides, due to a premature stop codon. ComF is a competence protein which is thought to play an important role in horizontal gene transfer and furthermore in developing multidrug resistance (67 , 68).

### Conclusions

The present study offers an unprecedented view of MRSA in India from isolates collected between 2014 and 2019 from 17 sentinel sites. Earlier studies have described two of the major dominant clones (ST772 and ST239) using WGS (19, 23, 69, 70, 42), and this is the first study that describes the third dominant clone (ST22) using this approach. In addition, the current study also offers details of less studied sequence types, such as ST2371, and provides a clear understanding of phylogenetic relationships between these. An in-depth analysis of the ST22 Indian isolates led to the discovery of two new lineages of hospital-acquired *Staphylococcus aureus* in India that are both PVL+ and carry resistance genes to fluoroquinolones and aminoglycosides. The presence of the *aac(*6′*)-aph(*2″) and *aadD* resistance genes in these lineages limits treatment options for patients, as these genes confer resistance to commonly used antibiotics. One of the lineages, IND-2, is particularly concerning as it also carries the *tsst-1* virulence gene, which increases the risk of severe infections such as toxic shock syndrome and necrotizing pneumonia (71). Hospital-acquired infections caused by these lineages can spread rapidly within healthcare settings, posing a risk to vulnerable patients.

Treating infections caused by these lineages may require more expensive antibiotics, longer hospital stays, and increased use of healthcare resources, leading to higher healthcare costs. Moreover, the geographic spread of these lineages, both within India and internationally, could pose a global public health threat. An analysis of these in a global context found evidence of protential transmission of the two Indian clones to other countries, i.e., the IND-1 cluster to the UK and to Italy and the IND-2 cluster to Russia.

It is important to continue monitoring and characterizing these lineages to inform public health interventions. The samples collected from various body sites and patients of all ages reflect the widespread nature of these lineages. The increased virulence and antimicrobial resistance of these lineages have contributed to their spread in India, making it crucial to identify and address them promptly. In the absence of WGS data, several previous studies have misidentified ST22 isolates as EMRSA-15; however, we were able to show that isolates with similar resistance and virulence profiles belong to the newly characterized Indian lineages.

The study also gives a comprehensive view of the ST2371, a sublineage of CC22, as a new emerging lineage in India and describes it in relationship with the other ST22 isolates. Thus, with the improved resolution afforded by WGS, this study substantially contributed to our understanding of the global population of MRSA. In addition, the retrospective identification of a putative outbreak of MDR ST239 from a single hospital in Bangalore that persisted over the period of 3 years highlights the need for the implementation of routine surveillance and simple infection prevention and control measures to reduce these outbreaks (72).

We acknowledge the limitations regarding the clinical information provided and the unknown clinical outcomes associated with the novel sublineages presented in this study, particularly when compared to ST772 or ST239. Furthermore, it is worth noting that the sampling strategies employed in this study resulted in an uneven representation of locations, time points, and infection types. Additionally, it is essential to recognize that the study does not encompass the entirety of India, indicating the need for expanding whole-genome sequencing-based surveillance to include more hospitals and provinces across the country. By doing so, a comprehensive understanding of the epidemiology and dynamics of *S. aureus* lineages in India can be achieved. This expansion will also facilitate the development of new diagnostic and typing methodologies to identify high-risk clones and enhance infection control measures.

## MATERIALS AND METHODS

### Bacterial isolates

A total of 508 retrospective clinical *S. aureus* isolates were obtained from 17 sentinel sites across 10 states of the country from the year 2014 to 2019 (Fig. 1; Table 1) through the network of sentinel hospitals established as previously described (73). Thirty genomes did not meet the quality criteria, and the remaining 478 isolates (393 MRSA and 85 MSSA) were included in the current analysis. Epidemiological metadata such as the age and sex of patients and infection type was collected by the sentinel sites. Isolates were confirmed as *S. aureus* at the reference laboratory morphologically and with the gram-positive ID card on Vitek-2 compact system (Biomerieux).

### Whole-genome sequencing, assembly, and annotation

DNA was isolated using the QIAamp DNA mini kit and quantified using Qubit as instructed by the manufacturer. Genome libraries with 450-bp insert size were prepared and sequenced on the Illumina platform with paired-end reads of 150-bp length. The data were assembled using the Spades assembler v.3.14 (74) to generate contigs and annotated with Prokka v.1.5 (75). Quality control of sequence data were performed using the GHRU quality control (QC) pipeline based on (i) the basic statistics of raw reads; (ii) the assembly statistics; (iii) contamination due to SNV and sequences from different species; (iv) species prediction using Bactinspector; and (v) overall QC as pass, warning, or fail of each isolate based on these different parameters as described in the pipeline (https://gitlab.com/cgps/ghru/pipelines/dsl2/pipelines/assembly/-/blob/master/qc_conditions_nextera_relaxed.yml). The assembly and other quality metrics of each isolate are provided in Table S11.

### Variant detection and phylogenetic analysis

The 478 isolates that passed QC were mapped to the reference genome of *S. aureus* strain MSSA476 (strain GCF_000011525.1, ST1) using the GHRU-SNP phylogeny pipeline v.1.2.2 (76). The mobile genetic elements (MGEs) were masked in the pseudo genome alignment using MGEmasker (77), and the recombination regions were removed using Gubbins v.2.0.0 (78). The nonrecombinant SNPs were utilized to build a maximum-likelihood tree using IQ-tree (79) with command line parameters “-czb” to collapse near-zero branches and a general time-reversible model with 1,000 bootstrap replicates. Visualization and phylogeographic analysis were performed on Microreact (80).

### Genotyping

ARIBA v.2.14.6 (81) and the PubMLST database were used for multilocus sequence typing of the isolates with the Oxford scheme using the seven housekeeping genes (*arcC*, *aroE*, *glpF*, *gmk*, *pta*, *tpi*, and *yqiL*) (https://pubmlst.org/organisms/staphylococcus-aureus). The goEBURST algorithm (82) in PHYLOViZ (83) software (83) was applied to assign STs to clonal complexes. Those sharing identical alleles at six of seven loci were interpreted as single-locus variants. From the ARIBA results, the isolates with ST designated as novel or ST* were further analyzed using the R package MLSTar (84) as described previously (85).

MLSTar queries the isolate sequence to reference the MLST database and assigns a novel allele sequence or the profiles associated with each housekeeping gene. The novel profile or the novel allele sequences identified by MLSTar were submitted to PubMLST, and new STs were assigned.

The spaTyper tool (86) was used to generate a *spa* type for the isolates based on the repeat sequences and repeat orders found on http://spaserver2.ridom.de/.

SCC*mec* typing was performed on the assembled contigs using Staphopia (60). Staphopia includes a primer-based SCC*mec* typing scheme where the primers are aligned using BLAST. In many cases, Staphopia failed to distinguish between SCC*mec* type V and SCC*mec* type VII because the identification of the SCC*mec* type is based on the *ccrC* genes. When more than one *ccrC* gene was present, e.g., both *ccrC1* and *ccrC2* genes are found, Staphopia reports the sample as “SCC*mec* V VII.” Thus, genomes with uncertain SCCmec types were resolved with an additional analysis with SCC*mec*Finder (87).

### Antimicrobial resistance determinants and virulence genes

Genome data were analyzed for the presence of virulence genes using the Virulence Factor Database (8). Resfinder (89) was used to identify acquired AMR genes in the genomes using the GHRU Resfinder pipeline (90) and mutations using the GHRU AMR pipeline (91) with the PointFinder database (92). All the bioinformatic analyses were performed using custom nextflow pipelines as detailed on protocols.io (93).

### Mobile genetic elements

Plasmid replicon types were identified with ARIBA v.2.10.1 (81) and the PlasmidFinder database (94). The 10 most prevalent plasmid replicon types in the entire data set and in the CC22 global collection are shown in the Microreact projects. A detailed list of all plasmid replicon types is shown in Tables S12 to S14.

### Nanopore sequencing and assembly

We sequenced representatives of isolates for which the SCC*mec*finder gave an alert as “Additional complex(es) was found” and also from the ST772 isolates which were found to carry *mecA* but no SCC*mec* cassette, using nanopore Minion-MK1C platform.

The same extracted DNA used for the Illumina sequence was used with the rapid barcoding kit SQK-RBK004 on FLO-MIN106 R9 flow cells to perform long-read sequencing. High-accuracy base calling was done with Guppy base caller v.4.3.4 used on MinKNOW v.21.02.20 (available at https://community.nanoporetech.com/downloads) to get fastq files, and the reads below q7 were filtered. Hybrid assembly was performed with both Illumina and nanopore reads using the nf-core Bacas assembly pipeline (95) v.1.1.1 (--assembler “unicycler”) to generate a single contig of the complete genome.

### CC22 global isolates

The 175 CC22 isolates from the current study were contextualised with 1623 CC22 isolates from previously published studies (Fig. 3A). Altogether, 1,408 genomes belonged to the EMRSA-15 clone and 105 belonged to the Gaza clone (Table 3; Table S5).

The midpoint-rooted phylogenetic tree of the CC22 global isolates was obtained from 44,703 SNPs, after mapping the genomes to the complete genome of strain EMRSA_15_GCF_000695215 and masking regions of recombination and MGEs.

A dated phylogenetic analysis was carried out using BactDating (47), using as an input file the output tree from Gubbins (78). We used the substitution rate as it was estimated for ST22 in a previous study (96). We could not establish the isolation year of two isolates (ERR234855 and ERR234880), which were thus excluded from the temporal analysis. The R code, the input and the output files of our analysis can be found in Supplementary Material 4 provided in Figshare (https://doi.org/10.6084/m9.figshare.21436047.v1).

## Data Availability

A list of the Microreact views referenced in this article is presented in Table S15. The sequencing data have been submitted to European Nucleotide Archive (ENA) under the project names PRJEB29740 & PRJEB50484. The metadata and other related data are available in the supplementary tables and in Microreact. Illumina read files of the strains used in the study have been deposited in European Nucleotide Archive, BioProject PRJEB29740. A full list of accession numbers for all sequence read files is provided in Table S5. Nanopore reads are submitted to ENA under the BioProject PRJEB50484. Metadata and other related information on the strains are provided in the Microreact project with different views in this link https://microreact.org/project/2xDvKQhriNveJ4kiVYsmSQ-s-aureus-wgs-study#i6az-ghru-tree. Strain information for the ST22 samples used from other studies is provided in Microreact at this link: https://microreact.org/project/2xDvKQhriNveJ4kiVYsmSQ-s-aureus-wgs-study#olhe-cc22-global-view-rectangular-tree and in Table S10.The authors confirm all supporting data, code and protocols have been provided within the article or the supporting data repository.

## SUPPLEMENTAL MATERIAL

Supplemental Material 1 msphere.00185-23-s0001.docx Legends to Figures S1 and S2; Tables S1-S4; legends to Tables S1-S15.

Supplemental Material 2 msphere.00185-23-s0002.xlsx Supplemental tables.

Supplemental Material 3 msphere.00185-23-s0003.xlsx Supplemental tables.

Figure S1 msphere.00185-23-s0004.pdf A simplified cartoon of an ACT pairwise comparison.

Figure S2 msphere.00185-23-s0005.pdf A simplified cartoon of an ACT pairwise comparison.

ASM does not own the copyrights to Supplemental Material that may be linked to, or accessed through, an article. The authors have granted ASM a non-exclusive, world-wide license to publish the Supplemental Material files. Please contact the corresponding author directly for reuse.
