# Supplementary material for: Novel multidrug-resistant sublineages of Staphylococcus aureus clonal complex 22 discovered in India
Source: mSphere. 2023 Sep 12;8(5):e00185-23. doi: 10.1128/msphere.00185-23 (PMC10597471; doi:10.1128/msphere.00185-23)
Supplement: Supplemental Material 1 — Legends to Figures S1 and S2; Tables S1-S4; legends to Tables S1-S15. [file msphere.00185-23-s0001.docx]

### Novel multi-drug resistant sub-lineages of *Staphylococcus aureus Clona*l Complex 22 discovered in India

Supplementary Figure 1: A simplified cartoon of an ACT pairwise comparison between the SCC*mec* cassette found in an Indian isolate (G18252308, (ST239, SCC*mec* marked as “IIIa*” in the Microreact) and the template sequences AB037671 (SCC*mec* type IIIa) and CP023560.1_NMR08 (SCC*mec* type III composite) shows that the SCC*mec* type found in G18252308 has lost the mer operon & pT181. It has a variant of the *pls* gene which is highlighted in green color.

####

Supplementary Figure 2: A simplified cartoon of an ACT pairwise comparison between the SCC*mec* cassette found in isolate G18255819 (ST22, IND-1 Custer, marked as SCC*mec* type IVc in Microreact) and the template sequence AB096217.1 (SCC*mec* type IVc) shows that the IND-1 isolate has been integrated with pUB110 plasmid which carries the genes *aadD1* (conferring resistance to Aminoglycosides) and *bleO* (conferring resistance to Bleomycin) highlighted in green color.

Supplementary table S1: Yearwise distribution of isolates collected across 17 different hospitals

| **Sentinel Site Code** | **2014** | **2015** | **2016** | **2017** | **2018** | **2019** | **Grand Total** |
| --- | --- | --- | --- | --- | --- | --- | --- |
| Hospital_1 |  |  |  |  | 3 |  | 3 |
| Hospital_2 |  |  |  |  | 4 |  | 4 |
| Hospital_3 |  |  | 11 | 30 |  |  | 41 |
| Hospital_4 |  |  |  |  | 10 |  | 10 |
| Hospital_5 |  |  |  |  | 1 | 4 | 5 |
| Hospital_6 |  |  |  |  | 19 | 10 | 29 |
| Hospital_7 |  |  | 30 | 50 | 44 |  | 124 |
| Hospital_8 |  |  |  |  |  | 10 | 10 |
| Hospital_9 |  |  | 9 |  |  | 10 | 19 |
| Hospital_10 |  |  |  |  |  | 11 | 11 |
| Hospital_11 |  |  |  |  | 5 |  | 5 |
| Hospital_12 | 60 | 48 | 53 |  |  | 10 | 171 |
| Hospital_13 |  |  |  |  |  | 6 | 6 |
| Hospital_14 |  |  |  |  |  | 1 | 1 |
| Hospital_15 |  |  |  |  | 6 |  | 6 |
| Hospital_16 |  |  |  |  | 9 |  | 9 |
| Hospital_17 |  |  |  |  | 19 | 5 | 24 |
| **Grand Total** | **60** | **48** | **103** | **80** | **120** | **67** | **478** |

Supplementary table S2: Yearwise distribution of MRSA & MSSA isolates collected from 2014 to 2019.

| **Count per year** | **2014** | **2015** | **2016** | **2017** | **2018** | **2019** | **Grand Total** |
| --- | --- | --- | --- | --- | --- | --- | --- |
| MSSA | 1 | 1 | 10 | 28 | 24 | 21 | 85 |
| MRSA | 59 | 47 | 93 | 52 | 96 | 46 | 393 |
| **Grand Total** | **60** | **48** | **103** | **80** | **120** | **67** | **478** |

Supplementary table S3: Count of isolates collected from different specimen sources

| **Specimen type** | **COUNT** |
| --- | --- |
| Axilla | 2 |
| Biopsy | 4 |
| Blood | 19 |
| Bone | 1 |
| Brain | 4 |
| Breast | 1 |
| Broncho-alveolar lavage | 1 |
| Catheter | 10 |
| Catheter central | 3 |
| Cerebrospinal fluid | 13 |
| Conjunctiva | 1 |
| Drain | 1 |
| Ear | 3 |
| Eyes | 3 |
| Fluid | 1 |
| Foot | 2 |
| Joint fluid | 2 |
| Nasopharynx | 1 |
| Pleural fluid | 1 |
| Pus | 274 |
| Shunt fluid | 7 |
| Sinus | 3 |
| Sputum | 5 |
| Throat | 2 |
| Tissue | 14 |
| Tracheal | 37 |
| Tracheal aspirate | 8 |
| Urine | 12 |
| Wound | 43 |
| **Grand Total** | **478** |

Supplementary table S4: Count of isolates as per gender and age distribution.

| **Age_Group (Year)** | **Female** | **Male** | **Grand Total** |
| --- | --- | --- | --- |
| <1 | 4 | 4 | 8 |
| 1-4 | 10 | 7 | 17 |
| 15-24 | 20 | 32 | 52 |
| 25-34 | 29 | 46 | 75 |
| 35-44 | 22 | 47 | 69 |
| 45-54 | 20 | 63 | 83 |
| 5-14 | 12 | 25 | 37 |
| 55-64 | 17 | 41 | 58 |
| 65-80 | 23 | 44 | 67 |
| >80 | 6 | 6 | 12 |
| **Grand Total** | **163** | **315** | **478** |

Supplementary table S5 (included in Supplementary Material 2): Metadata of the S. aureus collected in this study

Supplementary table S6 (included in Supplementary Material 2): Novel ST detected from the S. aureus collected in this study and the new ST assigned after submission to PubMLST database

Supplementary table S7 (included in Supplementary Material 2): AMR genes identified from the S. aureus collected in this study grouped as per the Antibiotic classes

Supplementary table S8 (included in Supplementary Material 2): Virulene genes detected from the S. aureus collected in this study grouped as per each virulence factor

Supplementary table S9 (included in Supplementary Material 2): Phages detected for each ST from the S. aureus collected in this study

Supplementary table S10 (included in Supplementary Material 3): Epidemiological and genomic characteristics of global ST22 isolates collected from 14 global studies

Supplementary table S11 (included in Supplementary Material 2): QC & Assembly statistics of the S. aureus collected in this study

Supplementary table S12 (included in Supplementary Material 2): Plasmid replicons identified from the S. aureus collected in this study

Supplementary table S13 (included in Supplementary Material 2): : Plasmid replicon cluster names for each cluster given by Ariba, for isolates from the GHRU collection

Supplementary table S14 (included in Supplementary Material 3): Plasmid replicon cluster names for each cluster given by Ariba, for isolates from the CC22 collection

Supplementary table S15 (included in Supplementary Material 3) Hyperlinks of Microreact views that describe the data collections presented in this study.
