# Supplementary figures and images for: Novel multidrug-resistant sublineages of Staphylococcus aureus clonal complex 22 discovered in India
Source: mSphere. 2023 Sep 12;8(5):e00185-23. doi: 10.1128/msphere.00185-23 (PMC10597471; doi:10.1128/msphere.00185-23)

Supplementary Figure 1 ST239

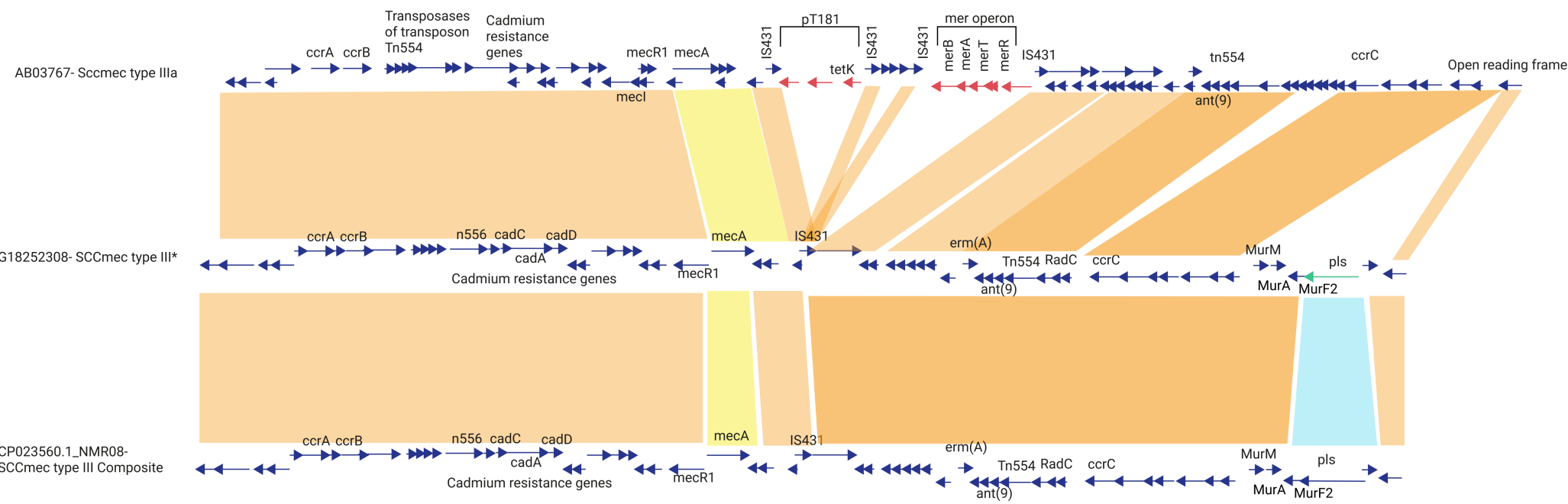

Supplement: Figure S1 — A simplified cartoon of an ACT pairwise comparison. [file msphere.00185-23-s0004.pdf]

Supplementary Figure 2 ST22

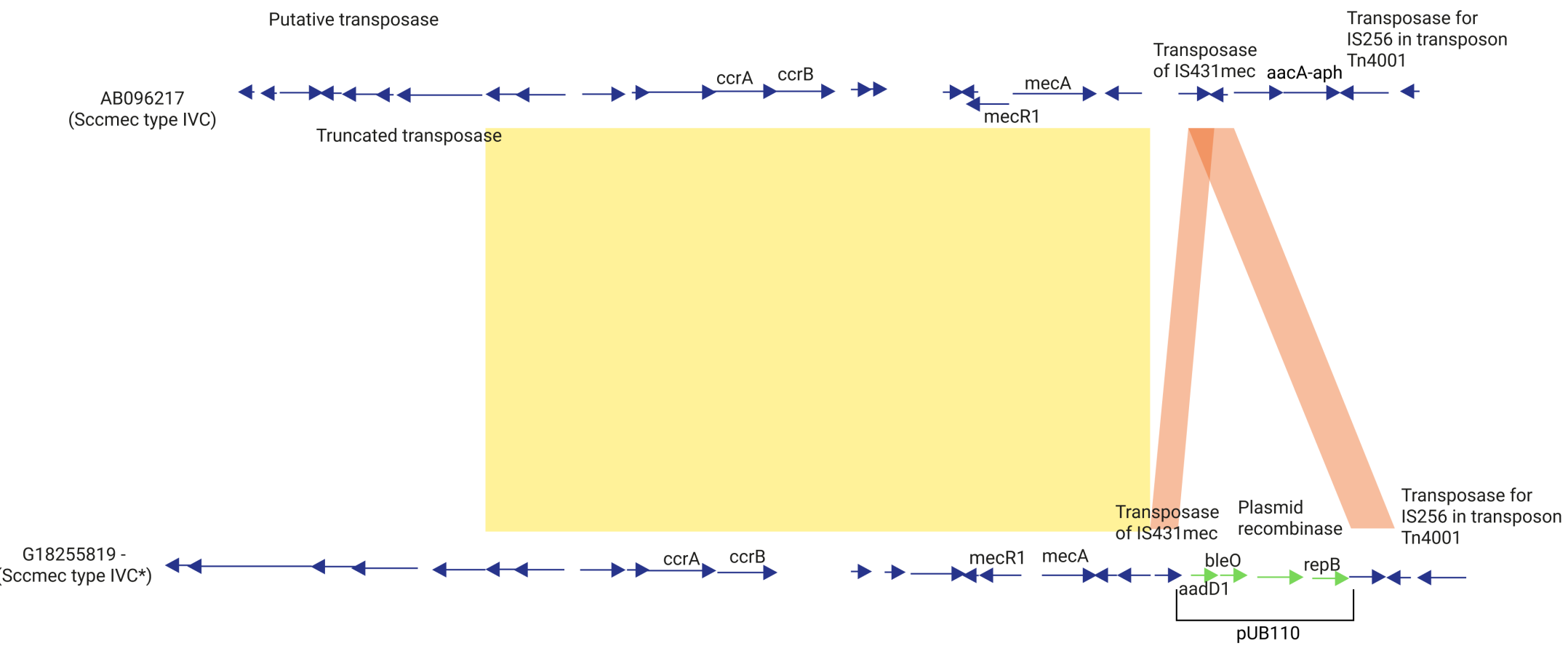

Supplement: Figure S2 — A simplified cartoon of an ACT pairwise comparison. [file msphere.00185-23-s0005.pdf]
